# Constrained VPH+: a local path planning algorithm for a bio-inspired crawling robot with customized ultrasonic scanning sensor

**DOI:** 10.1186/s40638-016-0043-1

**Published:** 2016-07-19

**Authors:** Akshay Rao, Mohan Rajesh Elara, Karthikeyan Elangovan

**Affiliations:** Temasek Laboratory, Singapore University of Technology and Design, 08 Somapah Road, Singapore, 487372 Singapore; Engineering Product Design Pillar, Singapore University of Technology and Design, 08 Somapah Road, Singapore, 487372 Singapore

**Keywords:** Biologically inspired robot, Multi-legged robot, Local path planner, Obstacle avoidance, Ultrasonic sensor

## Abstract

This paper aims to develop a local path planning algorithm for a bio-inspired, reconfigurable crawling robot. A detailed description of the robotic platform is first provided, and the suitability for deployment of each of the current state-of-the-art local path planners is analyzed after an extensive literature review. The Enhanced Vector Polar Histogram algorithm is described and reformulated to better fit the requirements of the platform. The algorithm is deployed on the robotic platform in crawling configuration and favorably compared with other state-of-the-art local path planning algorithms.

## Background

Robotic systems with the ability to reconfigure their morphologies in response to the application scenario display great potential with their versatility, fault tolerance, and efficiency for a variety of rugged missions in real world. A few works on reconfigurable robotics are [1–5].

Many reconfigurable robotic platforms are based on bio-inspired or biomimetic designs, which are based on naturally evolving mechanisms. A focus of reconfigurable robotic research has been the development of a bio-inspired platform which displays both rolling and crawling abilities, leading to the robotic platform known as BiLBIQ [6]. However, these efforts had been focused completely on mechanism design with almost no effort associated with perception or autonomous functionality.

While remote-controlled robot mechanisms have sufficed for most applications encountered thus far, emerging applications in the fields of surveillance and security necessitate the development of robots possessing a measure of autonomy and intelligence, including basic functionality such as mapping and local path planning. Unfortunately, integrating complex reconfigurable design mechanisms with perception introduces multiple new research challenges.

Recently, a family of reconfigurable robotic platforms (i.e., Scorpio) capable of crawling and rolling locomotion has been developed. These robots mimic the morphology of a huntsman spider that can transform between crawling and rolling by reconfiguring their legs.

The Scorpio is in development for applications pertaining to surveillance and security; thus, the primary focus has been the reduction in size of the platform, as well as improvement of power efficiency of the overall system. Furthermore, the changing requirements of the platform have resulted in a rapid evolution of five stages of the robotic platform in 2 years. In order to adhere to both these conditions, the Arduino Mini Pro 328 has been chosen as the onboard processing unit allowing for rapid prototyping of applications.

A customized ultrasonic sensor is designed for the system due to the relative computational inexpensiveness in processing the data, as well as to reduce power consumption. The customized sensor allows greater control of the sensor to restrict environmental observations to data that is relevant to the successful completion of the mission.

Previous works based on the Scorpio focus on the development of an efficient rolling controller [7, 8] and the formulation and implementation of an intelligent vision-based terrain perception module [9]. This paper attempts to add to the suite of autonomous functions for the Scorpio by developing an efficient obstacle avoidance algorithm in crawling mode.

One of the desired applications for the Scorpio platform has been the rapid traversal of unknown, unstructured terrain in a fixed period of time. A wall-following algorithm [10] was first implemented as a proof-of-concept obstacle avoidance algorithm, using the hardware described above. Despite its ability to traverse a room and find an exit, it was an inefficient algorithm, leading to oscillations in certain scenarios.

Conventional local path planning algorithms [11–22] assume the use of a robotic platform with Ackermann steering model, as well as highly accurate, rapidly scanning range sensors producing highly dense data. Since the Scorpio robot is unable to satisfy the assumptions for both the proprioceptive and the exteroceptive models, design of a local path planning algorithm that better fits the robot is necessitated.

In this paper, we present the design and development of a local path planning algorithm based on the Enhanced Vector Field Histogram (VPH+) and inspired by the inherent constraint due to the choice of sensor and platform. The remainder of the paper is organized as follows: The “Design of the robot” section describes the design specification of the robotic platform. Thereafter, in “Methods” section, we explain the design of the robot, sensor, and algorithm. The “Formulation” section derives the Constrained VPH+ algorithm from the VPH+ algorithm, while “Implementation” section provides a pseudocode for the algorithm, along with a discussion of some issues faced in implementing the algorithm on the Scorpio. The results are described in “Results” section. Finally, “Conclusions and future work” section concludes the paper and suggests avenues of future research.

### Huntsman spider

**Fig. 1:**
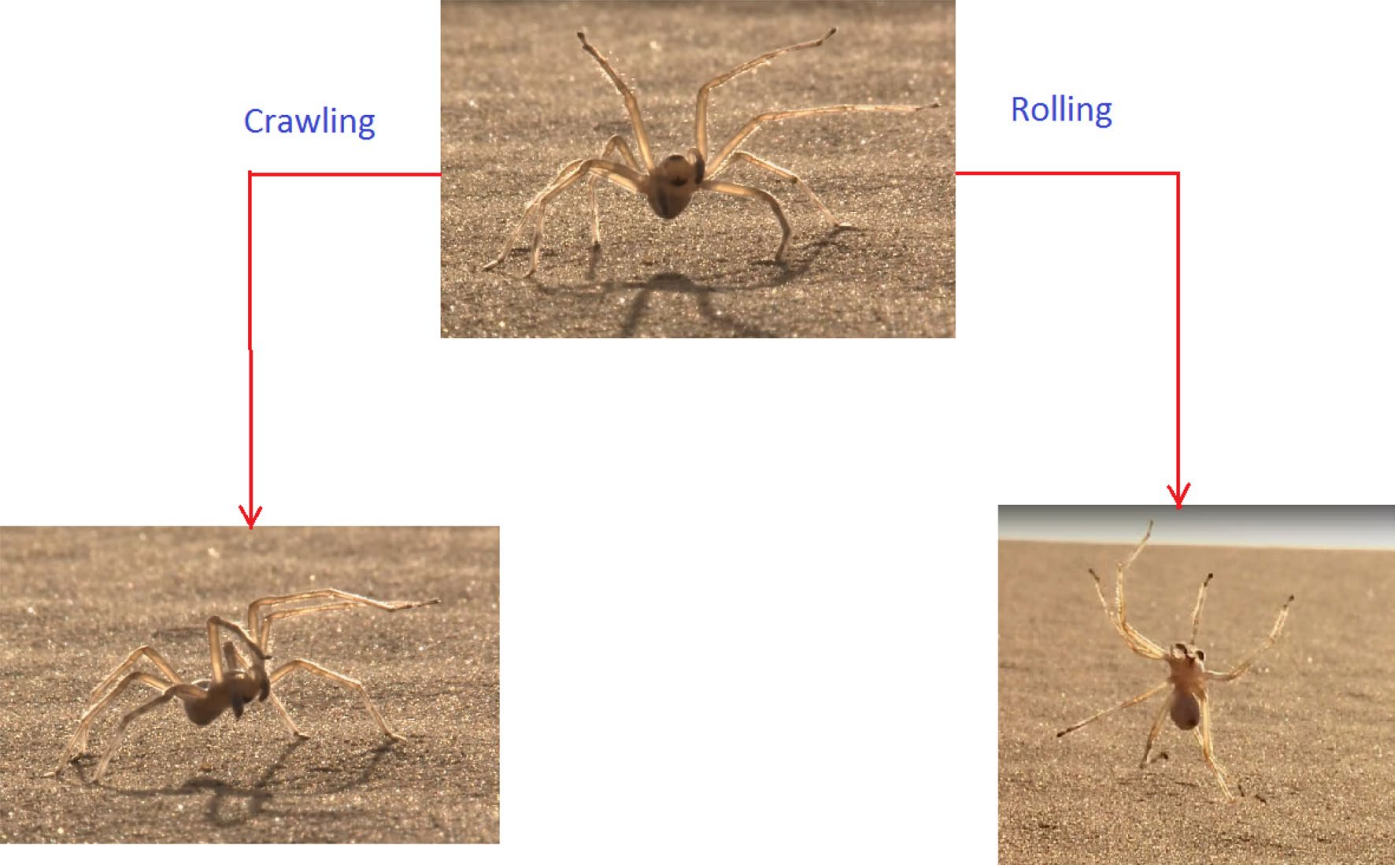
The huntsman spider. The huntsman spider of Southern Morocco (*top*) performing crawling motion (*bottom left*) and rolling motion (*bottom right*)

The Scorpio robot is inspired by the Cebrennus rechenbergi, a species of huntsman spider. This species is able to travel using both crawling and rolling motion, as shown in Fig. 1. The rolling locomotion of the huntsman spider was discovered by Ingo Rechenberg from TU Berlin [6]. The habitat of Cebrennus rechenbergi is the sand dunes of the Erg Chebbi desert in Southern Morocco on the boundary of the Sahara Desert.

While the spider normally crawls on eight legs similar to other species of spiders, when provoked or threatened by an external stimulus, it can escape by doubling its normal crawling speed using forward or backward flips with the use of its eight legs simultaneously, similar to acrobatic flic-flac movements used by gymnasts. Most notably, the spider turns somersaults to move independent of surrounding conditions. As a result, it does not need a slope to initiate the rolling process using the gravitational force. It also does not need to perform a run-up or a start-up gesture to trigger the rolling locomotion.

The main aim of the huntsman spider in deploying rolling mechanism as observed so far seems to maximize the displacement of the spider to escape from threatening circumstances, or when meeting a conspecific. So far, the spider has not been observed to perform any other functions in rolling mode, such as changing positions and hunting for prey.

### Local path planning

Local path planning and obstacle avoidance have been the subject of a great quantum of research in the robotics research community [11–22]. Initial papers focussed on development of techniques for perception and mapping using noisy sonar sensors [11–13].

An environment representation technique was arrived at in [12] known as the occupancy grid map, which is still used as a map representation technique in state-of-the-art algorithms. Elfes [13] first formulated the use of occupancy grids for navigation and mobile robot perception.

Borenstein and Koren [14] developed a local path planning algorithm known as Virtual Force Field (VFF), using occupancy grids for obstacle representation and potential fields [23] for navigation. The VFF algorithm employed the use of repelling force fields around obstacles and an attracting force in the direction of the target. The simplicity of the formulation made it an attractive path planning algorithm in the robotics research community.

Mathematical and practical drawbacks of the VFF were discovered and analyzed in [15]. While the occupancy grid method was a computationally inexpensive way to generate an approximate representation of the map, it was unable to compensate for the contradiction between the complexity and roughness of the grids, rendering it unsuitable for use with a low precision sensor like an ultrasonic range sensor. A tendency of the algorithm to get trapped in local minima was also discovered in scenarios where the goal was behind an obstacle.

In response to this problem, the Vector Field Histogram (VFH) was first introduced in [15] and further expanded and analyzed in [16, 17]. The VFH uses a two-dimensional Cartesian grid as a representative world model, while local environmental data are represented as a one-dimensional polar histogram around the robot. Each section of the polar histogram represents the obstacle density in that direction. The algorithm then chooses a direction which contains the best trade-off between the obstacle density and the goal direction. A subsequent step provided appropriate steering commands to generate motion in the desired direction.

Extensive testing and implementation of the VFH resulted in the discovery that the VFH algorithm does not take into account the vehicle kinematics, resulting in unrealistic and impractical inputs provided to the robot. In response, the Dynamic Window Planning approach was postulated in [18] which was specifically designed to account for the limited velocities and accelerations inherent in wheeled mobile robots.

The VFH was also improved, and its drawbacks addressed in the VFH+ algorithm [19]. The VFH+ algorithm added two extra stages to the VFH algorithm. The VFH algorithm also displayed the tendency of creating oscillations in environments with multiple narrow openings due to the sharp thresholding near the entrances. The first additional stage in the VFH+ algorithm was used to provide a hysteresis function between two threshold values, to reduce the oscillations and produce a smoother trajectory.

The next stage added to the VFH algorithm in the VFH+ algorithm was to mask the histogram representing obstacle density to consider only directions feasible for the robot to travel in. The robot was assumed to be a wheeled robot with Ackermann steering mechanism, and hence, the robot trajectory was assumed to be based on series of circular arcs.

The VFH* algorithm developed in [20] used the A* search algorithm to add look-ahead capability to the VFH+ algorithm to reduce problems arising from purely local obstacle avoidance.

An and Wang [21] developed the Vector Polar Histogram (VPH) algorithm by combining the VFH+ algorithm and the Potential Field Method. The VPH algorithm relied on the newly available laser range scanners with comparably higher accuracy to accurately represent the local obstacle map.

Gong et al. [22] extended the VPH algorithm to the VPH+ algorithm by grouping individual obstacles to obstacle blocks and determining concave blocks ahead of time, in order to increase the efficiency of the robot in traversing the environment. The VPH+ algorithm also extended the VPH cost function to take the robot heading and speed into account.

## Methods

### Design of the robot

The section presents the mechanical design and system architecture of the Scorpio robot.

### Mechanical design

The design of the Scorpio is based on the huntsman spider introduced in the previous section, to enable it to perform crawling and rolling locomotions. While the huntsman spider has eight legs, the Scorpio robot is designed with four legs which are adequate to perform crawling and rolling locomotions.

**Fig. 2:**
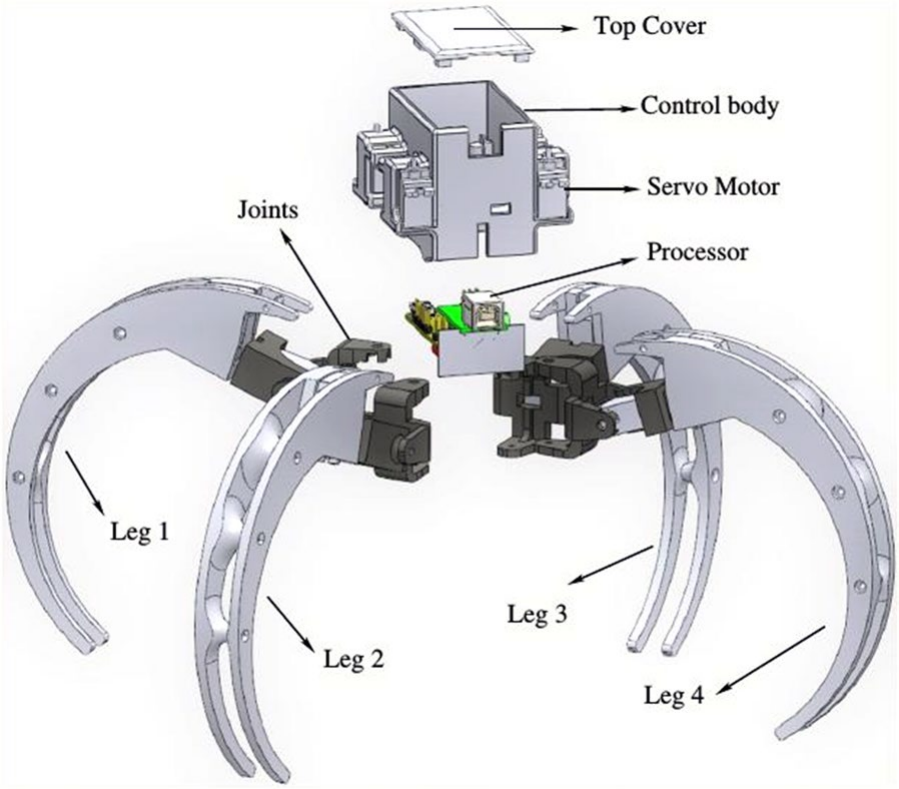
Exploded view of the Scorpio. An exploded view of the Scorpio model displaying all the components of the Scorpio

**Fig. 3:**
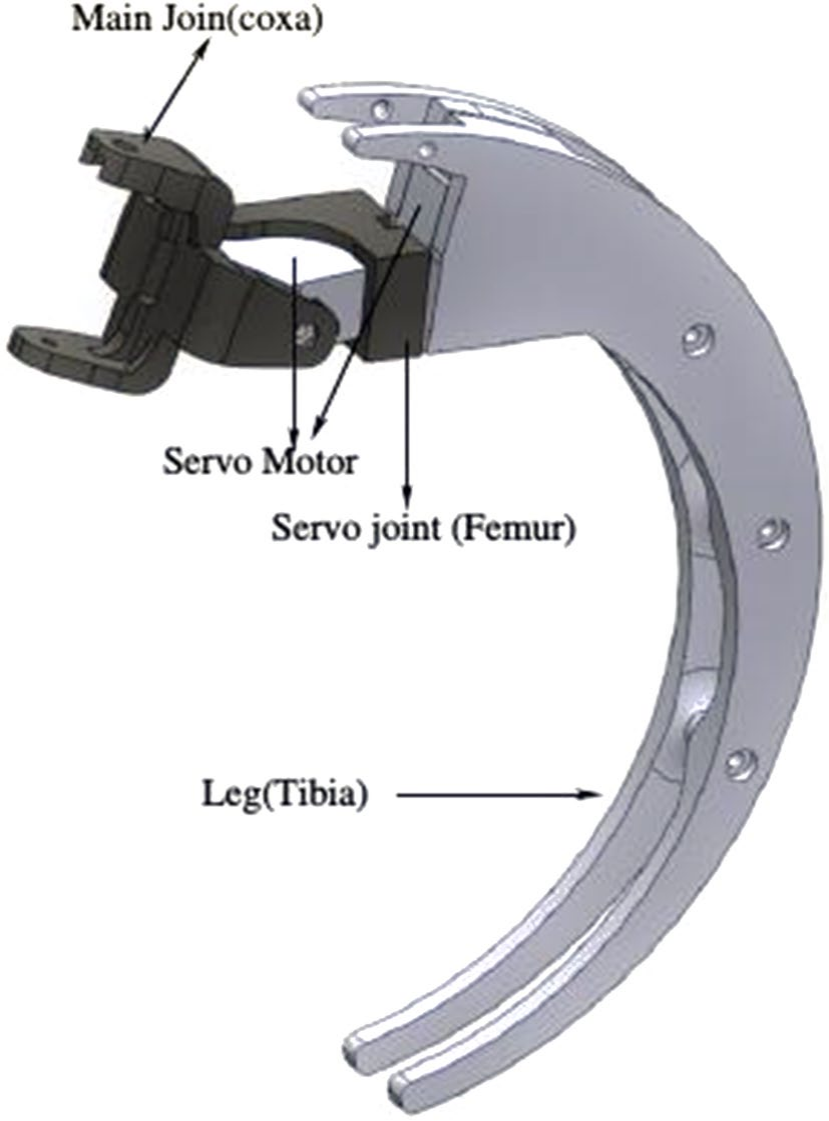
Scorpio leg. The model of an individual Scorpio leg

Figure 2 contains a part-by-part view of Scorpio robot showing the assemblies. It is observed that the Scorpio robot consists of four legs (tibia), four servo covers and joints (femur), four main joints (coxa), and a body. The processor, controller, and sensors are placed inside the body which is made from PLA plastic. Twelve servo motors are used in this Scorpio robot to generate locomotion. Each leg (model shown in Fig. 3) is mounted with three servos, so it has 3 degrees of freedom. These legs are able to rotate and transform from crawling to rolling gaits. The specifications of the Scorpio robot are listed in Table 1.

**Table 1:** Specifications of the Scorpio robot [24]

Controller	Arduino Mini Pro 328
Servo motor	JR ES 376
Servo controller	Pololu Micro Maestro 18 channel USB servo controller
Battery	LiPo 1200 mAh 7.4 v
ZigBee	XBee Pro S1, Digi International
Full body material	Polylactic acid (PLA)
Diameter (rolling) in mm	168 mm
LXWXH (walking) in mm	230 mm × 230 mm × 215 mm
Weight (full weight) in g	430 g

**Fig. 4:**
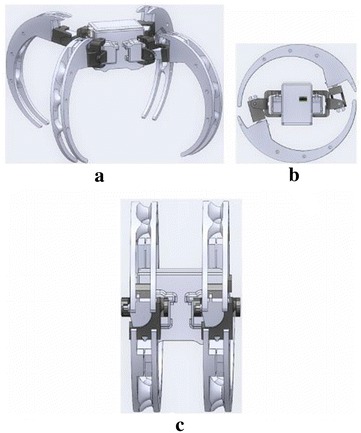
Scorpio robot model in different configurations. **a** Crawling configuration, **b** rolling configuration side view, **c** rolling configuration front view

For crawling motion, the Scorpio robot opens up its four legs as shown in Fig. 4a. The crawling involves 2 degrees of freedom. Transformation from crawling pose to cylindrical exoskeleton for rolling requires a motion of 3 degrees of freedom. The Scorpio robot uses its legs to push from the ground and shift the center of gravity to achieve the rolling motion with 1 degree of freedom. The rolling speed of the Scorpio robot doubles the rate of crawling speed.

### Sensor design

**Fig. 5:**
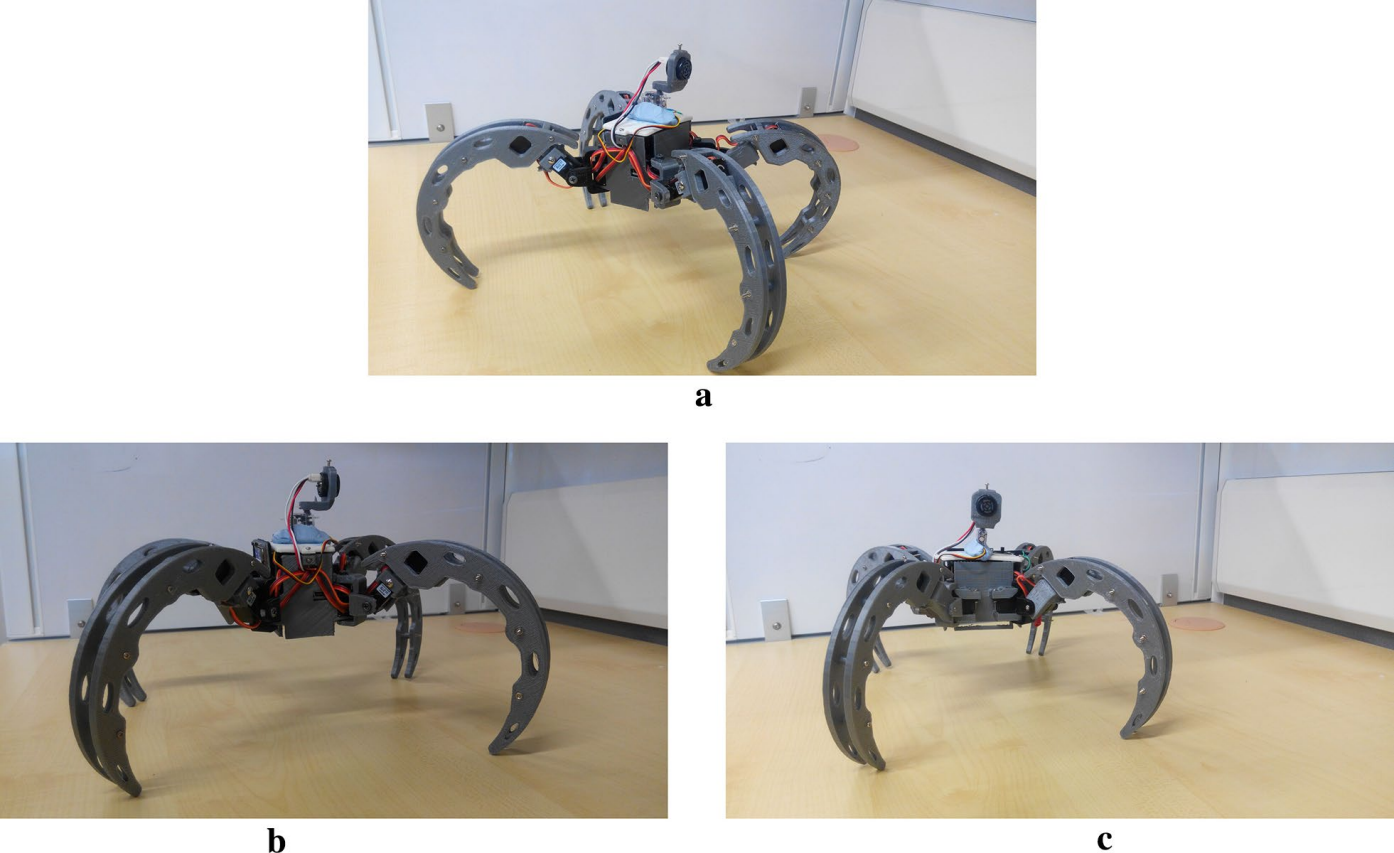
Scorpio with customized ultrasonic sensor. **a** Perspective view, **b** side view, **c** front view

The team aimed to create a customized range sensor with a greater degree of control. To this end, an ultrasonic sensor (model number SRF01) was mounted on a HS-35HD Ultra Nano Servo Motor. The ultrasonic sensor has a beam width of 12°. Further details of the sensor are tabulated in Table 2. A picture of the sensor mounted on the Scorpio can be found in Fig. 5.

**Table 2:** Specifications of the custom ultrasonic sensor

Sensor	SRF01
Beam width	10°
Minimum range	
Maximum range	6 m
Time period per data point	0.15 s
Motor	HS-35HD Ultra Nano Servo
Size in mm	18.6 × 7.6 × 15.5
Weight (g)	4.5
Torque (kg/cm)	0.8
Speed	0.10

**Fig. 6:**
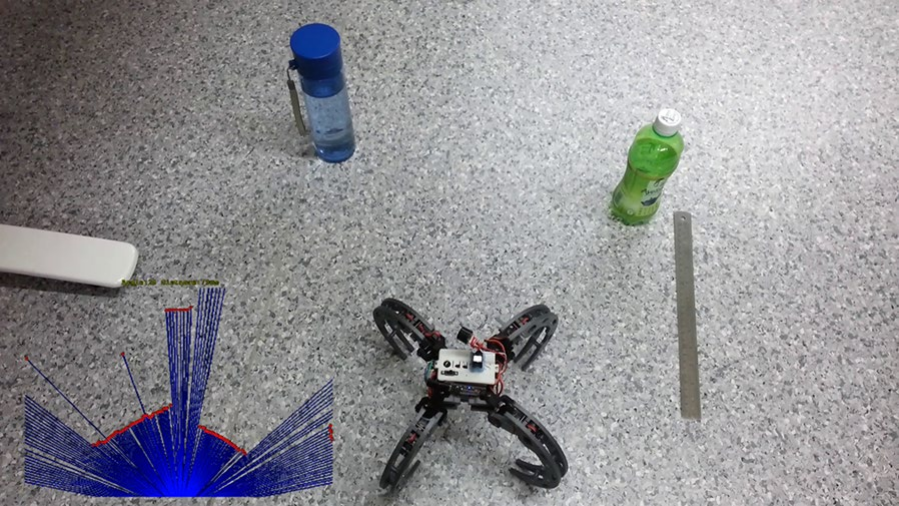
Sample ultrasonic scan. Sample scan with ultrasonic sensor

The servo motor is actuated to the desired angle, after which the ultrasonic sensor is triggered. The time taken for the sensor to rotate to the desired angle, transmit, and receive the data is roughly 0.15 s per point. Due to the slow speed of sensing, a comprehensive scan of the entire environment at each instance is not recommended. Figure 6 displays a sample scan taken with the ultrasonic sensor.

### Mode of locomotion

The Scorpio platform demonstrates different modes of locomotion in the two morphologies, each with their respective strengths and weaknesses.

- The rolling mode allows for greater speed of locomotion with reduced environmental perception.
- The crawling mode conversely trades better environmental perception for reduced locomotion.

This paper makes use of only the crawling mode of locomotion, to improve perception of environmental obstacles. While the limited field of view in rolling mode limits the potential for integration into the robot motion planner, future improvements in terrain traversal efficiency necessitate the use of rolling mode to be taken into account.

### Algorithm design

The local path planning algorithm design is informed by the following constraints inherent in the Scorpio robotic platform:

1. Kinematic constraints: Since the mode of locomotion in the Scorpio is crawling, standard wheeled robot kinematics are not valid. Consequently, all recent standard local path planning algorithms which assume Ackermann steering model cannot be used [14–22].
2. Sensor inaccuracy constraints: The customized ultrasonic sensor has a beam width of 15°, making it more inaccurate as the range increases. Thus, any obstacle in the sonar beam at the same range (as seen in Fig. 7) will produce the same reading. Hence, algorithms which rely on the accuracy of environmental information provided by the exteroceptive sensor [21, 22] will perform poorly on the Scorpio.

**Fig. 7:**
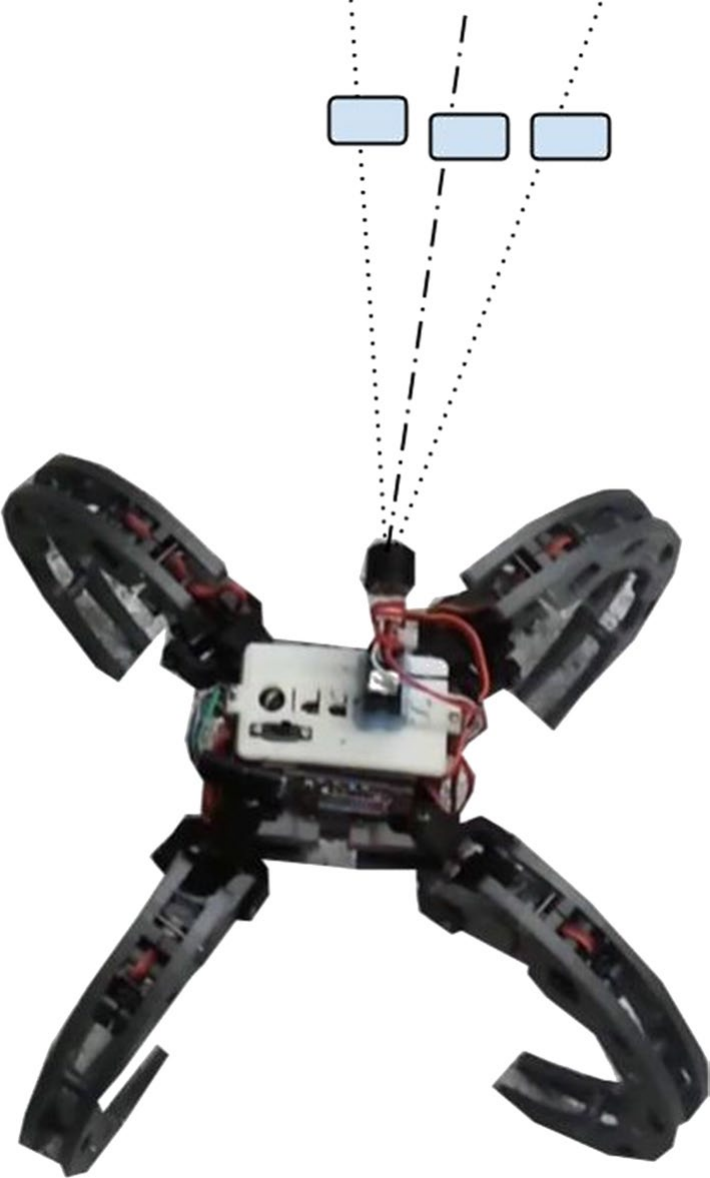
Inaccuracy due to beam width. All three obstacles at the same range will produce the same reading due to the large beam width
3. Sensor speed constraints: Slow scanning speed of the sensor implies performing scans over the full angular range frequently will negatively impact the efficiency of the algorithm. As a result, all recent local path planning algorithms which utilize the full sensor range [15–22] cannot be implemented.
4. Complex kinematic model: The Scorpio platform consists of twelve joint motors, resulting in a complex kinematic motion model. Computation of the entire kinematic model for locomotion on the Arduino processor used would greatly decrease speed and efficiency of the robot, rendering invalid all recent algorithms requiring computation of exact vehicle kinematics [18–22].
5. Processor constraints: The Arduino Mini Pro processor is single-threaded; thus, each separate action has to be performed serially. As a consequence, the platform is unable to scan the environment and move simultaneously resulting in the invalidation of all algorithms in which robot velocity is an important determinant of direction of motion [17–22].

Thus, to summarize, the optimal local path planning algorithm must be able to perform inferences on the local obstacle distribution using sparse, inaccurate data obtained at a low frequency. Furthermore, due to inefficiency of the mechanism while turning, it is desirable to ensure the robot motion is as straight as possible, with minimum turning. The goal of the algorithm is to ensure the robot traverses the maximum distance from the starting point in a fixed period of time.

The most recent state-of-the-art local path planning algorithms in the robotics research community are the Enhanced Vector Polar Histogram (VPH+) [22]. The VPH+ algorithm enhances the VPH algorithm formulation with obstacle grouping and classification, along with inclusion of the robot velocity and heading into the cost function. Since the sensors used by the Scorpio are too inaccurate to group obstacles accurately, and the single-threaded onboard processor ensures the platform will not be in motion when the algorithm is being computed, the VPH+ algorithm will be modified with suitable constraints to better fit the Scorpio platform. Motion primitives will be used to approximate the Scorpio kinematic model, to ensure acceptable performance on the Arduino Mini Pro processor.

## Formulation

The VPH+ algorithm represents the local environment as a polar histogram. It generates a cost function for each sensor angle using inputs from both the range sensor and the robot kinematic model. It leverages on the accuracy and the range of the laser range sensor to determine the boundaries of obstacle blocks, as well as to classify the obstacle as concave or convex. Concave obstacles are avoided outright, with the histogram value at the corresponding angles being set to zero.

### VPH+ algorithm

**Fig. 8:**
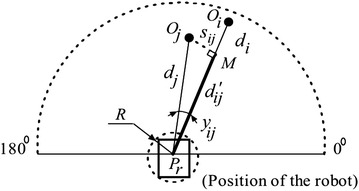
Grouping of obstacle blocks in VPH+. Adjacent points closer than a pre-defined threshold are classified as a single obstacle

Figure 8 depicts the diagram used to determine the reachable distance in each direction. The VPH+ algorithm modifies each range reading with the radius of the robot. Thus, in the diagram, the maximum traversable distance by the robot in direction *O*_*i*_ due to obstacle *O*_*j*_ is ${d'}_{ij}$, the length of $P_rM$ given by:

1 $${d'}_{ij} = \left\{ \begin{array}{ll} d_i & \quad s_{ij} > R\\ d_j \cos y_{ij} & \quad {\text {otherwise}} \end{array}\right.$$

where *y*_*ij*_ is the angle between points *i* and *j*, $s_{ij} = d_j \sin y_{ij}$, *R* is the robot radius.

The overall maximum traversable distance in direction *O*_*i*_ is given by

2 $$D_i = \min ({d'}_{ij}) - R; \quad (j=0,1,\ldots ,n-1)$$

where *n* is the total number of beams per scan given by $\pi / \alpha _{\mathrm{BW}}$ for a sensor with beam width *α*_BW_.

The observed points are then grouped as obstacles if the distance between them is lesser than a user-defined safe threshold *d*_thr_, which is larger than the robot radius, plus a buffer distance. The distance between two adjacent points is given by:

3 $$d_{i,i+1} = \sqrt{{d_i}^2 + {d_{i+1}}^2 - 2d_id_{i+1} \cos \alpha _{\mathrm{BW}}}$$

The VPH+ classifies obstacles as concave or convex, depending on which the following symbol function is constructed:

4 $$B(i) = \left\{ \begin{array}{ll} 0 & \quad i \in {\text {concave obstacle block}}\\ 1 & \quad {\text {otherwise}} \end{array}\right.$$

The kinematics of the robot are then taken into consideration with the current robot velocity, minimum turning radius, and maximum robot velocity being used to generate a value for the safe distance *d*_safe_. A threshold function *H*(*i*) similar to *B*(*i*) is then created such that

5 $$H(i) = \left\{ \begin{array}{ll} 1 & \quad {D_i \ge d_{\mathrm{safe}}}\\ 0 & \quad {\text {otherwise}} \end{array}\right.$$

**Fig. 9:**
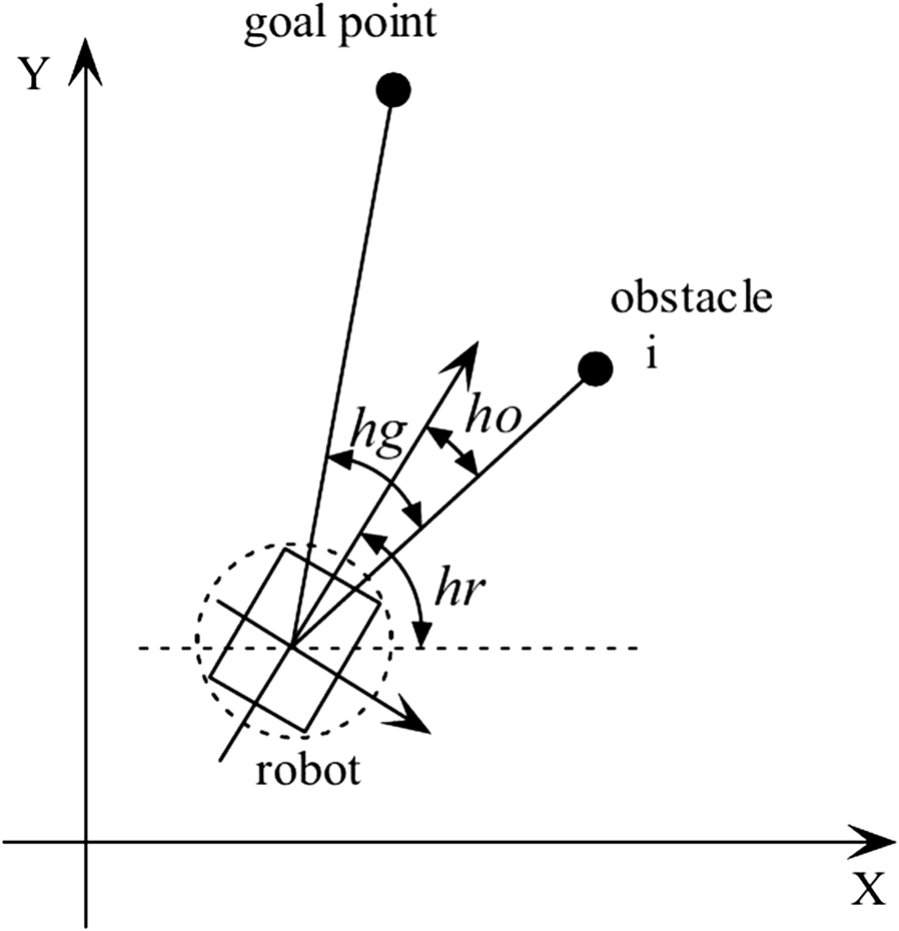
Calculation of cost function in VPH+. Angles used for the calculation of each cost function

An angular cost function is constructed considering the angles shown in Fig. 9 given by:

6 $$S(i) = k_1h_{\mathrm{g}} + k_2h_0 + k_3$$

where *h*_g_ is the angle between the goal and the current sensor angle, *h*_0_ is the angle between the current direction of the robot and the current sensor angle, *k*_1_, *k*_2_, and *k*_3_ are user-defined constants, $k_1h_{\mathrm{g}}$ is the cost associated with deviating from the direction to the goal, $k_2h_0$ is the cost associated with deviating from the current direction of motion, and *k*_3_ is a nonzero constant used to ensure the denominator is nonzero. Thus, a robot with high *k*_1_ and low *k*_2_ will prioritize goal following at the cost of maintaining a smooth trajectory.

The final cost function is calculated with the individual cost functions with the expression:

7 $$C(i) = \frac{B(i)H(i)D_i}{S(i)}$$

The desired direction is the direction with the maximum cost function.

8 $$\theta _{\mathrm{final}} = \max (C(i))$$

### Effect of sensor inaccuracy

**Fig. 10:**
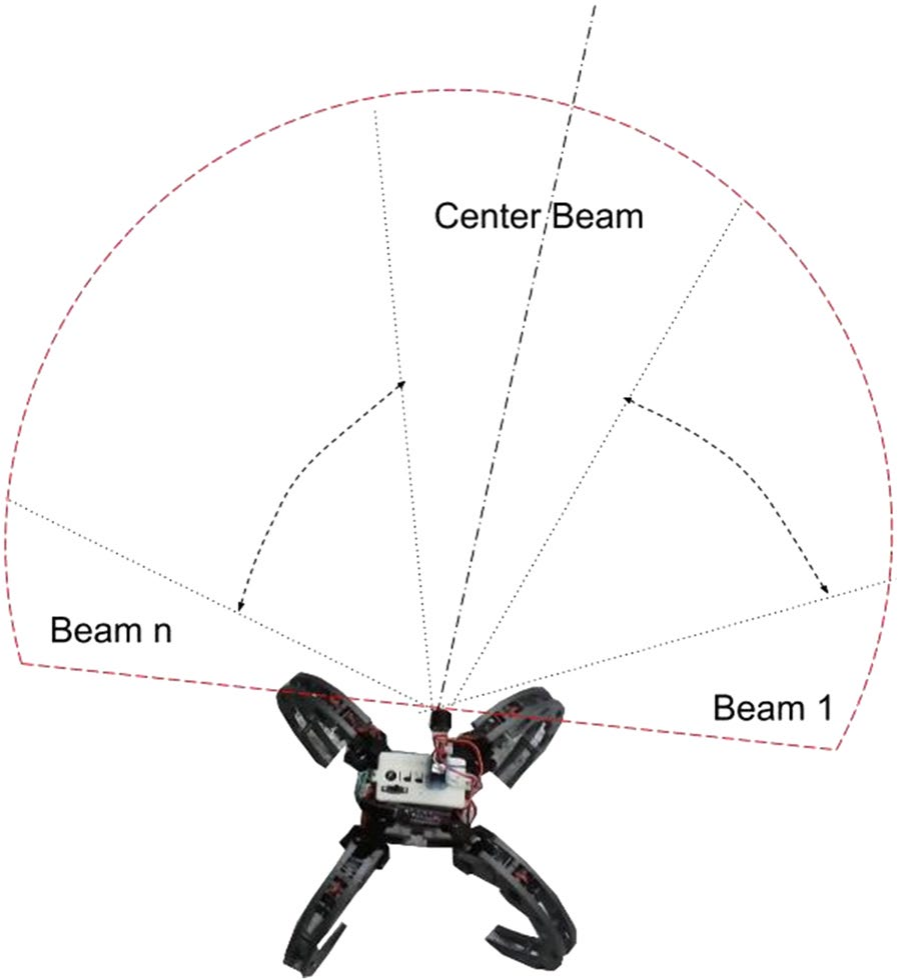
Sensor field of view. Field of view of the custom-made ultrasonic sensor used

The design of the Constrained Enhanced Vector Polar Histogram (CVPH+) is informed by the constraints from the inaccuracy of the sonar sensor, the small size of the platform, as well as the low processing power of the onboard processing unit. The field of view of the ultrasonic sensor can be divided into *n* sectors, as shown in Fig. 10, where *n* is given by:

9 $$n = \frac{\alpha _{\mathrm{FOV}}}{\alpha _{\mathrm{BW}}}$$

where *α*_FOV_ is the total field of view of the sensor and *α*_BW_ is the beam width of the sensor, as defined earlier.

**Fig. 11:**
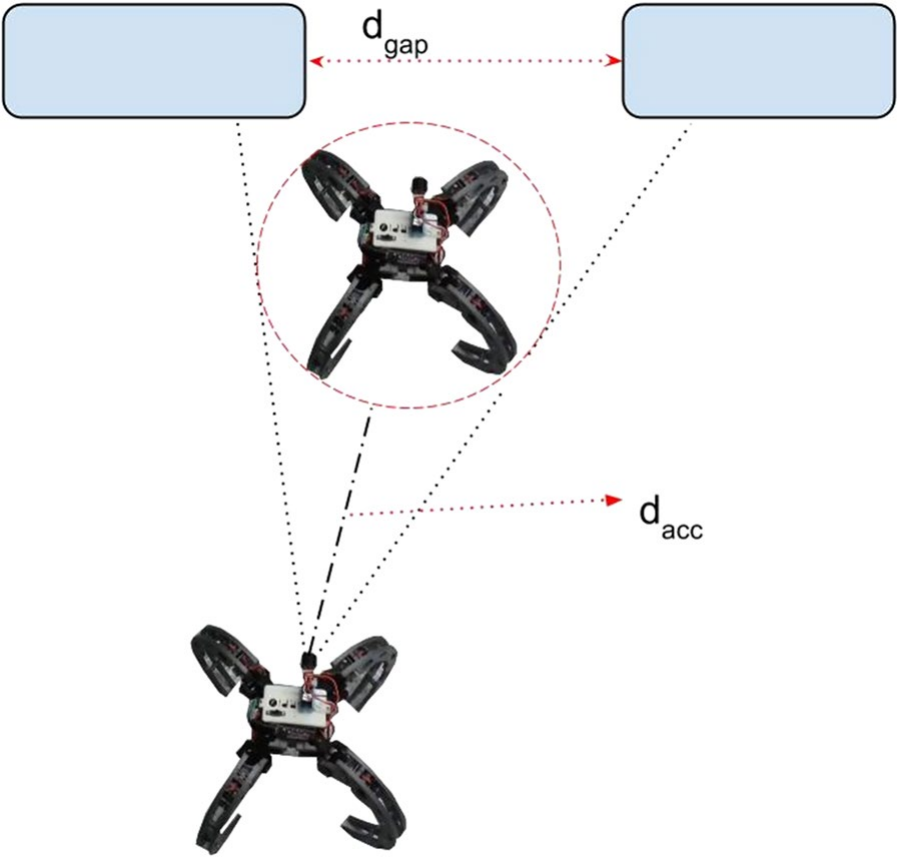
Sensor inaccuracy. Constraints to the algorithm due to inaccuracy of the sensor

Inaccuracies arising due to the beam width of the sonar are of the order of the robot size. For example, consider the scenario in Fig. 11, in which acceptable distance *d*_acc_ is the range at which the lateral beam width becomes equal to the diameter of the robot (*d*_R_, say). The value for *d*_acc_ can be found from the expression given by:

10 $$d_{\mathrm{acc}} = \frac{d_{\mathrm{R}}}{\alpha _{\mathrm{BW}}}$$

Consequently, obstacle pings with ranges greater than *d*_acc_ cannot be used directly in VPH+ for obstacle grouping and to obtain obstacle boundaries. When the range reading from obstacles is less than *d*_acc_ (implying that the distance between two obstacles *d*_GAP_ is less than the size of the robot *d*_R_), further accuracy regarding obstacle spacing becomes unnecessary; hence, the ranges can be used directly to obtain further inferences regarding the obstacle field.

In the case of [22], the range sensor used was the LMS200, which has an angular beam width of 4.4 millirad [25] and a maximum range of 80 m. Thus, the diameter of the robot must be greater than 0.352 m, according to Eq. 10. The Pioneer P3AT robot used by them has a width of 0.381 m, which fulfills this criterion.

### Cost function reformulation

In the cost function for the VPH+ algorithm, the *B*(*i*) and *H*(*i*) terms are indicator terms having values of only 1 or 0. Consider the modified cost function

11 $$C'(i) = \frac{k_1h_{\mathrm{g}} + k_2h_0}{D_i}$$

Assuming the same values for *B*(*i*) and *H*(*i*) in the VPH+ formulation, in this case, the desired direction at the end of the inference cycle will be given by:

12 $$\theta _{\mathrm{final}} = \min (C'(i))$$

Let the absolute angle for the goal as seen in Fig. 9 be *α*_*G*_ and the absolute angle for the point *i* be *α*_*i*_. Then, from Fig. 9, the following relations are true:

13 $$h_{\mathrm{g}}= \alpha _G - \alpha _i$$

14 $$h_0= h_r - \alpha _i$$

The cost function then becomes:

15 $$C'(i) = \frac{k_1(\alpha _G - \alpha _i) + k_2(h_r - \alpha _i)}{D_i} = \frac{K_{\alpha } - K_k \alpha _i}{D_i}$$

where $K_{\alpha }$ is a constant for the current inference cycle given by:

16 $$K_{\alpha } = k_1\alpha _G + k_2h_r$$

and *K*_*k*_ is a constant for the algorithm obtained from the following expression:

17 $$K_k = k_1+k_2$$

Another angle, *α*_*j*_, will be chosen if its cost function $C'(j)$ will be lesser than $C'(i)$. However, the maximum range of the sensor *r*_max_ implies that an upper limit to the angle can be determined and is given by the expression:

18 $$\alpha _{j,\max } \le \frac{K_k}{K_{ \alpha }} - \frac{r_{\mathrm{max}}}{D_iK_{\alpha }}(K_{\alpha } - K_{k}\alpha _i)$$

By observation, it can be seen that the numerator of the cost function is minimum for scan angles that directly point toward the goal or toward the robot heading, depending on the values of *k*_1_ and *k*_2_.

Hence, the maximum scanning angle for the current scan needs to take both into account, along with the maximum possible angle reachable by the sensor $\alpha _{\mathrm{sensor,max}}$.

19 $$\alpha _{\mathrm{max}} = \max ( \alpha _{G,\max }, \alpha _{0,\max }, \alpha _{\mathrm{sensor,max}})$$

The resulting number of beams required is then obtained by:

20 $$n_s = \frac{ \alpha _{\mathrm{max}}}{ \alpha _{\mathrm{BW}}}$$

A simple example would be when the robot is pointed toward the goal (i.e., $\alpha _G = h_r$, and there is no obstacle in front). In this case, it is expected that the cost function will be minimum in the direction of the goal; hence, no other angle can have a cost function lower than the goal direction. Sure enough, on inputting the values in the above equation, we receive

21 $$\alpha _{j,\max } \le \alpha _G$$

Since *α*_*G*_ is the first angle sector, the relation simplifies to $\alpha _{j,\max }$ being equated to *α*_*G*_, the goal angle.

**Fig. 12:**
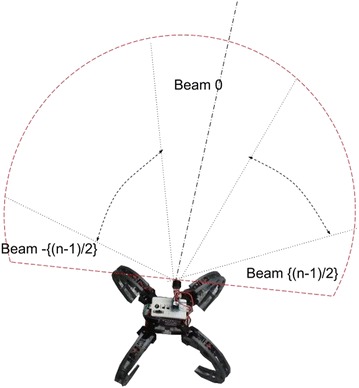
Sonar sector numbering. Numbering convention used for sonar data

Figure 12 shows the numbering convention used for the sonar data. The sonar scan is divided into an odd number of scans *n*. The center beam is numbered 0, the beams on the left are numbered negative, while the beams on the right are numbered positive.

## Implementation

**Fig. 13:**
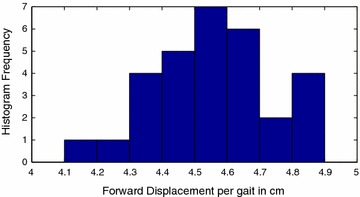
Forward motion gait primitive. Histogram for forward motion gait primitive

**Fig. 14:**
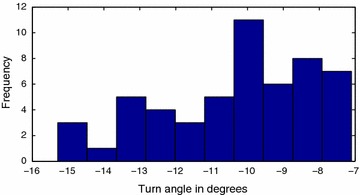
Leftward motion gait primitive. Histogram for leftward motion gait primitive

**Fig. 15:**
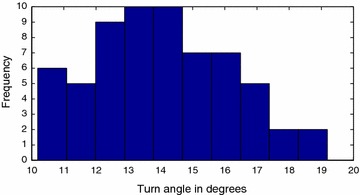
Rightward motion gait primitive. Histogram for rightward motion gait primitive

Gait primitives for the Scorpio robot were first obtained. The motion of the Scorpio robot in three directions was characterized: left, right, and forward. Histograms of the gaits were obtained, as shown in Figs. 13, 14 and 15.

**Fig. 16:**
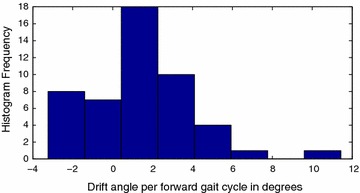
Forward motion drift. Histogram for forward motion drift

Structural inaccuracies introduced due to the inexact nature of the 3D printing process resulted in different values for left and right gait primitives. The forward motion gait was also discovered to display a drift, as shown in Fig. 16, which was compensated for in the implementation of the algorithm.

The values of *k*_1_ and *k*_2_ were obtained from by optimizing the implementation of the VPH+ algorithm for the best result, as tabulated in Table 3.

**Table 3:** Parameters used in experiment

*α*_BW_	12°
*r*_max_	8 m
*r*_min_	0.18 m
*α*_FOV_	165°
*n*_BEAM_	15
*d*_*R*_	0.15 m
*d*_acc_	1.15 m
*k*_1_	0.6
*k*_2_	0.4

The pseudocode of the Constrained VPH+ algorithm is displayed below.
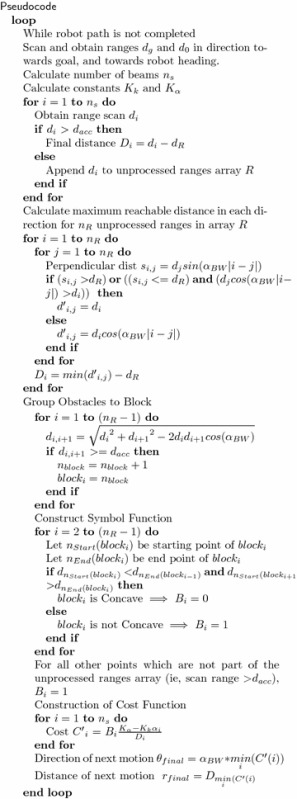


**Fig. 17:**
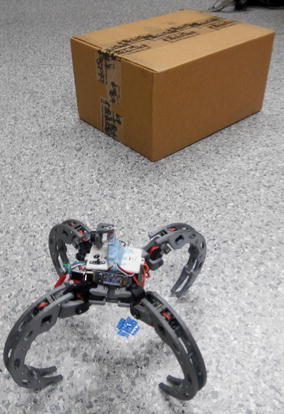
Simple obstacle course. Simple obstacle course for the robot

**Fig. 18:**
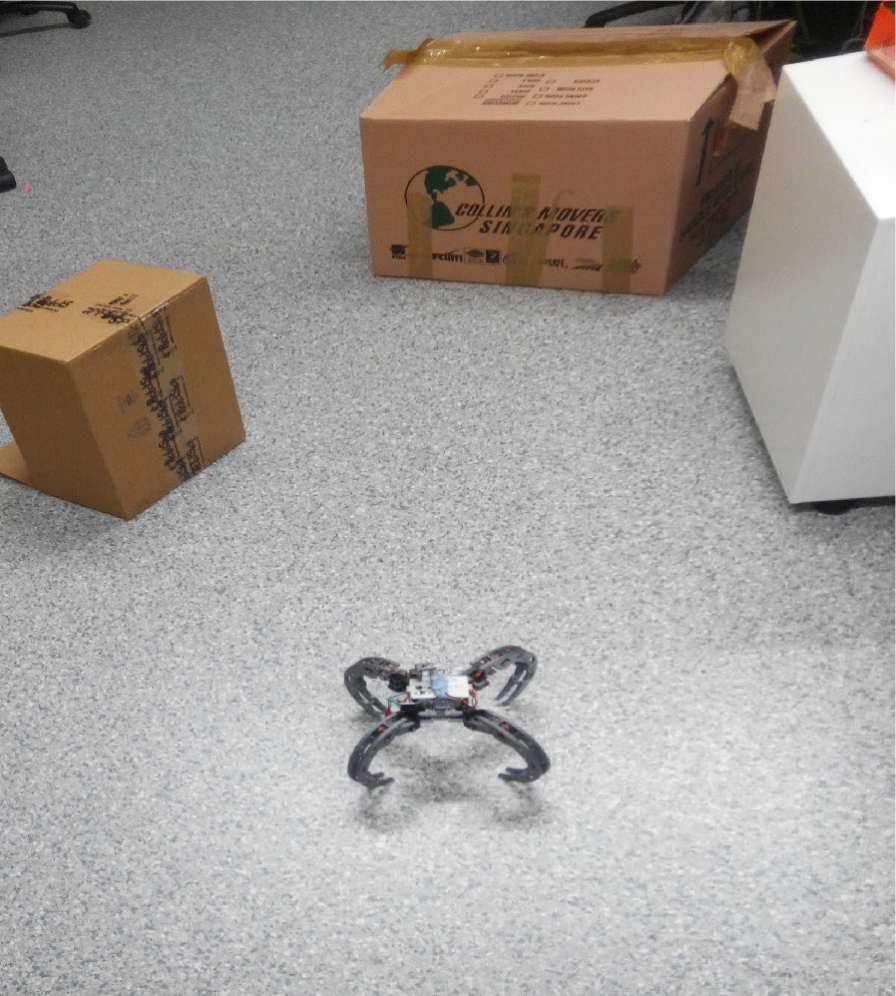
Simple obstacle course path comparison. Path comparison in simple obstacle course

The algorithm and its performance were compared against the VPH+ algorithm on two obstacle courses shown in Figs. 17 and 18. First, a simple obstacle course was created consisting of a single obstacle, on which the algorithms were evaluated. A second, more complicated obstacle course was created with a higher obstacle density.

The height of the ultrasonic sensor is around 17 cm from the ground; hence, it was attempted to ensure obstacles of a similar minimum height. The cluttered office environment is used to ensure effectiveness of the algorithm in realistic settings.

To ensure minimal errors due to pose inconsistency, the goal was set to be simply 1.20 m from the start point. The trials were conducted 6 times for each algorithm at each obstacle course.

## Results

**Fig. 19:**
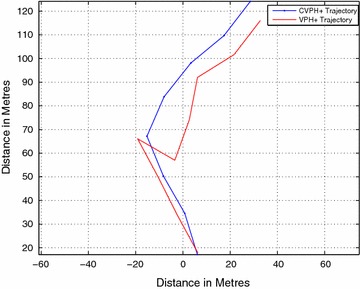
Crowded obstacle course. Crowded obstacle course for the robot

Figure 19 shows the comparison of the paths taken by the robot for the simple obstacle course, consisting of a single obstacle, shown in Fig. 17. As seen in the figure, the paths taken by the robot due to both the algorithms compare very closely. However, the VPH+ algorithm causes the robot to double back due to erroneous grouping of obstacle caused by the wide beam width of the sonar.

The VPH+ algorithm took an average of 249.7254 s, while the CVPH+ algorithm clocked an average time of 224.3952 s.

**Fig. 20:**
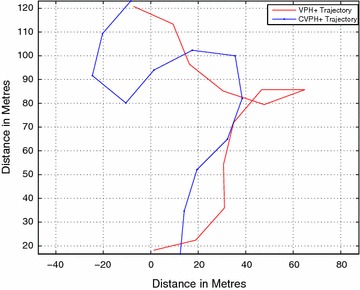
Crowded obstacle course path comparison. Path comparison in crowded obstacle course

Figure 20 shows the comparison between the paths taken in the more complicated obstacle course shown in Fig. 18. The closeness of the two obstacles in comparison with sonar beam width causes the VPH+ algorithm to erroneously group them together, resulting in the robot doubling back halfway to look for another path. The CVPH+ algorithm, however, closely follows the contours of two obstacles, resulting in a longer, more conservative path. The cause of this path is the choice of constants *k*_1_ and *k*_2_, which were optimized for the first obstacle course. Further optimization of the constants can lead to more performance gains for the CVPH+ algorithm.

The VPH+ algorithm took an average of 336.5960 s, while the CVPH+ algorithm took 329.4032 s.

## Conclusions and future work

The CVPH+ algorithm is found to outperform the VPH+ algorithm in both empty and clutter-filled environments. The improvements in the algorithm were obtained from formalizing implicit assumptions made in the VPH+ algorithm regarding the sensor and the robot model, both of which were invalid for the Scorpio robot.

Further insight was obtained from examining the original VPH+ cost function further and reformulating it to improve the performance of the CVPH+ algorithm.

Pose inaccuracy due to the noisy gait primitive estimates, as well as the inherent inaccuracies due to the 3D printing process, has been seen to reduce the performance of both the VPH+ and the CVPH+ algorithms. A more comprehensive formulation taking into account pose and map estimates will result in better performance of the algorithms.

A further avenue of future research includes the use of the rolling configuration of the robot to improve its speed in traversing the environment. The challenges caused by the displacement of the ultrasonic sensors in the rolling mode of the robot will also be an interesting aspect to be explored.

## References

[1] Nansai S, Rojas N, Elara MR, Sosa R. Exploration of adaptive gait patterns with a reconfigurable linkage mechanism. In: 2013 IEEE-RSJ international conference on intelligent robots and systems (IROS). IEEE, Japan. 2013; p. 4661–8.

[2] Yim M, Shen W-M, Salemi B, Rus D, Moll M, Lipson H, Klavins E, Chirikjian GS (2007). Modular self-reconfigurable robot systems [grand challenges of robotics]. IEEE Robot Autom Mag.

[3] Murata S, Kurokawa H (2007). Self-reconfigurable robots. IEEE Robot Autom Mag.

[4] Moubarak P, Ben-Tzvi P (2012). Modular and reconfigurable mobile robotics. Robot Auton Syst.

[5] Gilpin K, Kotay K, Rus D (2008). Miche: modular shape formation by self-disassembly. Int J Robot Res.

[6] King RS. BiLBIQ: a biologically inspired robot with walking and rolling locomotion, biosystems and biorobotics, vol. 2. Springer. 2013. http://www.springer.com/engineering/robotics/book/978-3-642-34681-1.

[7] Nemoto T, Mohan RE, Iwase M (2015). Realization of rolling locomotion by a wheel-spider-inspired hexapod robot. Robot Biomim.

[8] Nemoto T, Mohan RE, Iwase M (2015). Energy-based control for a biologically inspired hexapod robot with rolling locomotion. Digit Commun Netw.

[9] Sinha A, Tan N, Mohan RE (2014). Terrain perception for a reconfigurable biomimetic robot using monocular vision. Robot Biomim.

[10] Dwyer B. Wall following robot. Diss. Worcester Polytechnic Institute; 2013.

[11] Elfes A. A sonar-based mapping and navigation system. In: Proceedings 1986 IEEE international conference on robotics and automation, vol. 3. 1986; p. 1151. doi:10.1109/ROBOT.1986.1087534.

[12] Elfes A. Occupancy grids: a probabilistic framework for mobile robot perception and navigation. 1989.

[13] Elfes A (1989). Using occupancy grids for mobile robot perception and navigation. Computer.

[14] Borenstein J, Koren Y. High-speed obstacle avoidance for mobile robots. In: Proceedings of the 1988 IEEE international symposium on intelligent control. 24–26 Aug 1988; p. 382, 384. doi:10.1109/ISIC.1988.65461.

[15] Koren Y, Borenstein J, Potential field methods and their inherent limitations for mobile robot navigation. In: Proceedings of the 1991 IEEE international conference on robotics and automation, vol. 2. 9–11 Apr 1991; p. 1398, 1404. doi:10.1109/ROBOT.1991.131810.

[16] Borenstein J, Koren Y. Real-time obstacle avoidance for fast mobile robots in cluttered environments. In: Proceedings of the 1990 IEEE international conference on robotics and automation, vol. 1. 13–18 May 1990; p. 572, 577. doi:10.1109/ROBOT.1990.126042.

[17] Borenstein J, Koren Y (1991). The vector field histogram-fast obstacle avoidance for mobile robots. IEEE Trans Robot Autom.

[18] Fox D, Burgard W, Thrun S (1997). The dynamic window approach to collision avoidance. IEEE Robot Autom Mag.

[19] Ulrich I, Borenstein J. VFH+: reliable obstacle avoidance for fast mobile robots. In: Proceedings of the 1998 IEEE international conference on robotics and automation, vol. 2. 16–20 May 1998; p. 1572, 1577. doi:10.1109/ROBOT.1998.677362.

[20] Ulrich I, Borenstein J. VFH*: local obstacle avoidance with look-ahead verification. In: 2000 Proceedings of the ICRA ’00 IEEE international conference on robotics and automation, vol. 3. 2000; p. 2505, 2511. doi:10.1109/ROBOT.2000.846405.

[21] An D, Wang H (2004). VPH: a new laser radar based obstacle avoidance method for intelligent mobile robots. IEEE Int Conf Intell Control Autom.

[22] Gong J, Duan Y, Man Y, Xiong G. VPH+: an enhanced vector polar histogram method for mobile robot obstacle avoidance. In: ICMA 2007. International conference on mechatronics and automation, 2007. 5–8 Aug 2007; p. 2784, 2788. doi:10.1109/ICMA.2007.4304000.

[23] Khatib O. Real-time obstacle avoidance for manipulators and mobile robots. In: Proceedings of the 1985 IEEE international conference on robotics and automation, vol. 2. Mar 1985; p. 500, 505.

[24] SICK AG. Telegrams for operating/configuring the LMS 2XX laser measurement systems. Firmware version V.2.10/X1.14. Sick AG: Waldkirch, Germany. 2003.

[25] Lanz-Cortiella R, Llorens-Calveras J, Rosell-Polo JR, Gregorio-Lopez E, Palacin-Roca J (2011). Characterisation of the LMS200 laser beam under the influence of blockage surfaces. Influence on 3D scanning of tree orchards. Sensors.

